# Triple Layer Tungsten Trioxide, Graphene, and Polyaniline Composite Films for Combined Energy Storage and Electrochromic Applications

**DOI:** 10.3390/polym12010049

**Published:** 2019-12-30

**Authors:** Hailong Lyu

**Affiliations:** 1 Chemical Sciences Division, Oak Ridge National Laboratory, Oak Ridge, TN 37831, USA; hlyu@utk.edu; 2 Department of Chemistry, University of Tennessee, Knoxville, TN 37996, USA

**Keywords:** polyaniline, WO_3_, graphene, electrochromic, electrochemical energy storage

## Abstract

Different polyaniline (PANI)-based hybrid films were successfully prepared by electro-polymerizing aniline monomers onto pre-spin-coated indium tin oxide (ITO) glass slides with WO_3_, graphene, or WO_3_/graphene films. Comparing with pristine PANI, the shifts of the characteristic peaks of PANI-based nanocomposites in UV-visible absorption spectra (UV-vis) and Fourier transform infrared spectroscopy (FT-IR) indicate the chemical interaction between the PANI matrix and the nanofillers, which is also confirmed by the scanning electron microscope (SEM) images. Corresponding coloration efficiencies were obtained for the WO_3_/PANI (40.42 cm^2^ C^−1^), graphene/PANI (78.64 cm^2^ C^−1^), and WO_3_/graphene/PANI (67.47 cm^2^ C^−1^) films, higher than that of the pristine PANI film (29.4 cm^2^ C^−1^), suggesting positive effects of the introduced nanofillers on the electrochromic performance. The areal capacitances of the films were observed to increase following the order as bare WO_3_ < WO_3_/graphene < pristine PANI < WO_3_/PANI < graphene/PANI < WO_3_/graphene/PANI films from both the cyclic voltammogram (CV) and galvanostatic charge-discharge (GCD) results. The enhanced energy storage and electrochromic performances of the PANI-based nanocomposite films can be attributed to the capacitance contributions of the introduced nanofillers, increased PANI amount, and the rougher morphology due to the embedment of the nanofillers into the PANI matrix. This extraordinary energy storage and electrochromic performances of the WO_3_/graphene/PANI film make it a promising candidate for combined electrochromic and energy storage applications.

## 1. Introduction

Energy shortage and environmental pollution have been two major subjects of modern society, which urgently requires developing clean, efficient, and renewable sources of energy, as well as advanced technologies associated with energy storage and conversion [1,2,3]. As a promising novel energy storage device with fast charging-discharging rate and extremely long cycling life, electrochemical supercapacitor possesses not only higher energy density than conventional dielectric capacitors but also higher power density than common batteries [4,5]. Electrochemical supercapacitors include two typical categories based on different charge storage mechanisms, that is, electric double-layer capacitors (EDLCs) with a non-Faradic process through the ion adsorption between the interfaces of electrodes and electrolyte, and pseudo-capacitors with a Faradic process via fast surface redox reaction [6]. Carbon materials usually employed as EDLCs exhibit excellent cycling life (>10^5^ cycles) but limited capacitance. In contrast, metal oxides and conducting polymers as main materials for pseudo-capacitors always possess much larger capacitances but the shrinkage and swelling lead to much shorter cycling life. In order to overcome their corresponding deficiencies, it is proposed that the combination of EDLCs materials with pseudo-capacitive materials is an excellent choice since this approach can take advantage of both the long cycling life of EDLCs and high capacitance merits of metal oxides [7].

Meanwhile, electrochromism (EC) has also attracted intense research attention over the last few decades, where the material color changes with electrochemical reactions [8,9]. The persistent but reversible color change of electrochromic materials can be easily controlled by a temporarily employed electrical potential [10], showing wide potential applications such as smart windows, display devices, vehicle sunroofs, and antiglare mirrors for cars [11,12,13]. For example, the light transmittance or reflectance properties of electrochromic windows can be adjusted by voltage through reversible lithium intercalation, allowing the controllable heat transfer and lighting conditions [14]. Various types of materials have been reported to show electrochromic properties, such as transition metal oxides, mixed-valence materials, organic molecules, and conjugated polymers [15,16,17]. 

Among all the materials for supercapacitor and electrochromic applications, conducting polymers belonging to both categories have received a lot of research interests because of their tunable electrical properties, flexibility, and high processability within solution [18]. Among all the polymers, solution-processable conductive polymers can be proceeded through a low-cost approach to fabricate thin-film electrodes, offering adjustable thickness, high conductivity, excellent optical transparency, and superior electrochromism [19,20]. Particularly, polyaniline (PANI) has attracted extensive attention by the good electrochemical and thermal stabilities, tunable properties, low-cost, and high conductivity [21,22]. The pseudo-capacitance and corresponding color change from the various redox reactions make PANI a promising candidate for combined electrochemical capacitor and electrochromic applications [23]. However, multiple electrochromic materials or “multicoloring” have to be applied to increase the range of available colors and reproduce high-quality electrochromism in such devices. One straightforward method is to mix two discrete colors, which can be achieved by applying two kinds of electrochromic materials with complementary colors deposited onto two different working electrodes [12,24,25]. Furthermore, the switching rate of electrochromic films could be improved by faster ion transport since it is a prerequisite for electrochromic materials to maintain electroneutrality that ions be transported into and out of the film during redox reactions [26]. Therefore, uniform and tunable porous structure is a beneficial feature enabling facile ion transport and redox-active films [8]. Nowadays, tungsten trioxide (WO_3_) as a typical electrochromic material with certain pseudocapacitive property have been widely investigated and demonstrated promising application potentials for electrochromic and supercapacitor electrodes [27,28,29,30]. Therefore, the combined WO_3_ with PANI is expected to give multicolored and enhanced energy storage performances due to the combined electrochromic and pseudocapacitive properties as well as the resulted porous morphology. However, since PANI and WO_3_ exhibit bad cycling stabilities during the redox reaction [31,32], carbon materials are always integrated into the nanocomposites because of their excellent cycling stability [33,34,35,36]. Graphene with the two-dimensional (2D) carbon structure can well meet the requirement owing to its unique electrical, chemical, thermal, and mechanical properties [37,38,39]. Especially, graphene has been demonstrated to stabilize the conducting polymer by forming a certain relationship thus enhancing the conductivity, energy storage ability, and cycling stability of pristine polymers [40,41,42,43]. All in all, the introduction of either WO_3_ or graphene is expected to have positive effects on the electrochromic and supercapacitor performance of the pristine PANI films. 

In this work, we have successfully synthesized different hybrid WO_3_, graphene, and PANI films by employing combined spin coating and electro-polymerization method. The interactions between the specific components and the morphologies of the synthesized different hybrid films (bare WO_3_, pristine PANI, double layer WO_3_/graphene, double layer WO_3_/PANI, double layer graphene/PANI, and triple layer WO_3_/graphene/PANI films) have been successfully investigated by various characterization methods. Their energy storage and electrochromic performances were also studied. All the PANI-based hybrid films exhibit greatly enhanced energy storage abilities and coloration efficiencies compared to the pristine PANI, demonstrating positive effects of the nanofillers. The triple layer WO_3_/graphene/PANI film shows great potential for combined energy storage and electrochromic applications. To the best of our knowledge, comprehensive characterizations and studies of different hybrid WO_3_, graphene, and PANI films for energy storage and electrochromic applications have not been reported.

## 2. Materials and Methods

### 2.1. Materials

Aniline (C_6_H_7_N, ≥99.0%), sulfuric acid (H_2_SO_4_, 95.0–98.0%), hydrogen peroxide solution (PERDROGEN® 30% H_2_O_2_ (w/w)), ethanol (HPLC, 99.8%), and ammonium hydroxide (NH_4_OH, 28.86 wt %) were all purchased from Fisher Scientific (Hampton, VA, USA). Tungsten oxide (WO_3_, 99.9+%, 40–50 nm, Stock # 5506BD, CAS # 1314-35-8, Lot # 5506-021115) were purchased from Nanostructured & Amorphous Materials, Inc. (Katy, TX, USA). Graphene was provided by Celtig LLC (Knoxville, TN, USA). The microscope glass slides and indium tin oxide (ITO) coated glass slides were also obtained from Fisher Scientific (Hampton, VA, USA). Before the usage of the ITO coated glass slides, they were first sonicated in ethanol for 10 min, and then immersed in an aqueous solution containing 4.0 mL NH_4_OH, 4.0 mL H_2_O_2_ and 20.0 mL deionized water for 10 min. Finally, the ITO glasses were sonicated in deionized water for another 10 min and dried naturally. Deionized water was used throughout the experiments.

### 2.2. Thin Film Electrode Preparation

Six species of films as bare WO_3_, double layer WO_3_/graphene, pure PANI, double layer graphene/PANI, double layer WO_3_/PANI, and triple layer WO_3_/graphene/PANI were synthesized through employing a combined spin coating and electro-polymerization method, respectively. For the synthesis of WO_3_ film, 5.0 mg WO_3_ was dispersed in 10.0 mL ethanol solution under 30 min sonication. The WO_3_ film was prepared by drop casting about 1.0 mL WO_3_ suspension onto the ITO glass and maintained at 2000 rpm for 20 s. WO_3_/graphene film was synthesized by continued drop casting another 1.0 mL graphene suspension (5.0 mg graphene dispersed in 10.0 mL ethanol solution undergoing 30 min sonication) on the synthesized WO_3_ film and maintained at 2000 rpm for another 20 s. The electro-polymerization of aniline onto the as-treated ITO glass or formed WO_3_, graphene, or WO_3_/graphene films were performed on an electrochemical working station VersaSTAT 4 potentiostat (Princeton Applied Research, Oak Ridge, TN, USA). A typical three electrode electrochemical cell was employed, in which a saturated calomel electrode (SCE) served as the reference electrode, a platinum (Pt) mesh served as the counter electrode and the spin coated ITO glass or bare ITO glass slide with an effective area of 6.0 cm^2^ served as the working electrode. A typical electrochemical polymerization was performed 10 cycles scanned back and forth from 0 to +1.2 V vs. SCE at a scan rate of 50 mV/s in 0.5 M H_2_SO_4_ aqueous solution containing 0.5 M aniline. All the films were dried naturally overnight.

### 2.3. Characterizations

The morphologies of the thin films grown on the ITO glass slides were characterized by scanning electron microscope (SEM, Hitachi S4300, Tokyo, Japan) and atomic force microscopy (AFM, Agilent 5600 system with multipurpose 90 mm scanner, Santa Clara, CA, USA). The thickness of the thin films grown on the ITO glass slides were also determined by AFM. Imaging of AFM was done in acoustic ac mode (AAC) using a silicon tip with a force constant of 2.8 N/m and a resonance frequency of 70 kHz. The FT-IR spectrometer coupled with an ATR accessory (Vector 22, Bruker Inc., Billerica, MA, USA) was used to characterize the surface functionality of the thin films grown on the ITO glass slides in the range of 2000 to 500 cm^−1^ at a resolution of 4 cm^−1^. The UV-vis of the films deposited on the ITO coated glass slide were observed in the range of 200–800 nm at room temperature using a UV-visible spectrophotometer (Jasco V-670 spectrophotometer and spectralon was used as a reference, Easton, MD, USA). A long path length home-made spectro electrochemical cell with Teflon cell body with front and rear windows clapped with two steel plates was used where the ITO glass slide was used as the working electrode for energy storage and optical characterizations. In a standard three-electrode system, the electrochemical behaviors of the PANI-based nanocomposites films were investigated by CV scanned from −0.2 to 0.8 V vs. SCE at a series of scan rates and galvanostatic charge-discharge measurements from 0 to 0.8 V with different current densities. The electrochemical impedance spectroscopy (EIS) was carried out in the frequency range from 100,000 to 0.01 Hz at a 5 mV amplitude referring to the open circuit potential (OCP) on the same electrochemical working station VersaSTAT 4 potentiostat. The cycling stabilities of the PANI-based films were also investigated by evaluating the capacitance retentions by running 1000 galvanostatic charge-discharge cycles. The spectroelectrochemistry measurements were performed on a Jasco V-670 spectrophotometer coupled with the potentiostat for applying electrochemical potentials. The in situ chronocoulometry (CC) were conducted under a square-wave voltage of 0.8 and −0.2 V with a pulse width of 20 s using the electrochemical working station VersaSTAT 4 potentiostat. 

## 3. Results and Discussion

### 3.1. Thin Film Electrode Preparation

**Figure 1:**
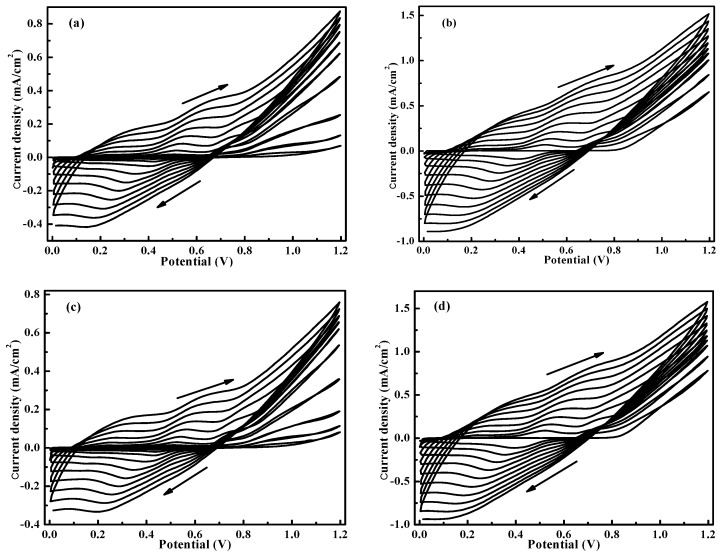
Electro-polymerization synthesis of polyaniline (PANI) onto (**a**) bare indium tin oxide (ITO) glass, (**b**) graphene-coated ITO glass, (**c**) WO_3_-coated ITO glass, and (**d**) double layer WO_3_/graphene-coated ITO glass at a scan rate of 50 mV/s in 0.5 M H_2_SO_4_ aqueous solution containing 0.5 M aniline.

Figure 1 shows the potentiodynamic electro-polymerization growth of PANI onto the (a) bare ITO, (b) graphene-coated ITO, (c) WO_3_-coated ITO, and (d) double layer WO_3_/graphene-coated ITO. The PANI film are deposited on the glass by sweeping the potential between 0 and 1.2 V at a scan rate of 50 mV/s in 0.5 M H_2_SO_4_ aqueous solution containing 0.5 M aniline. The film growth can be clearly verified by the continuously increased current with the CV cycles. Similar CV curves except different anodic current peaks are obtained for these PANI-based nanocomposites films. The anodic irreversible peaks start at around +0.9 V, corresponding to the oxidation of aniline monomers and the initiation of the PANI electro-polymerization. However, it is noted that the anodic peak current densities of graphene/PANI (Figure 1b, 1.53 mA/cm^2^) and WO_3_/graphene/PANI (Figure 1d, 1.55 mA/cm^2^) are much higher than that of pristine PANI (Figure 1a, 0.88 mA/cm^2^), which can be attributed to the improved electrical conductivity caused by the introduced graphene. In contrast, WO_3_/PANI shows decreased anodic peak current density (Figure 1c, 0.76 mA/cm^2^) compared to pristine PANI film because of the increased resistance of WO_3_ film on the ITO.

In addition, the mass of the electropolymerized PANI onto the substrate can be roughly estimated through the total Faradic charges consumed in the electro-polymerization assuming that 2.5 electrons for each aniline monomer in emeraldine, as the following Equation (1):

*m* = *CM_m_*/2.5*F* (1)

where *m* is the mass of PANI polymerized onto the substrate, gram (g); *C* is the total Faradic charges consumed in the electro-polymerization, coulomb (C); *M_m_* is the molecular mass of aniline monomers (93.13 g/mol); and *F* is Faraday constant (96,485 C/mol). About 14.6, 28.6, 12.3, and 27.8 μg PANI polymers were calculated for the pristine PANI, WO_3_/PANI, graphene/PANI, and WO_3_/graphene/PANI films, respectively. The fewer and higher amount of PANI in the WO_3_/PANI and graphene/PANI films further confirm the increased and decreased resistance caused by the introduced WO_3_ and graphene, respectively.

### 3.2. Materials Characterization

**Figure 2:**
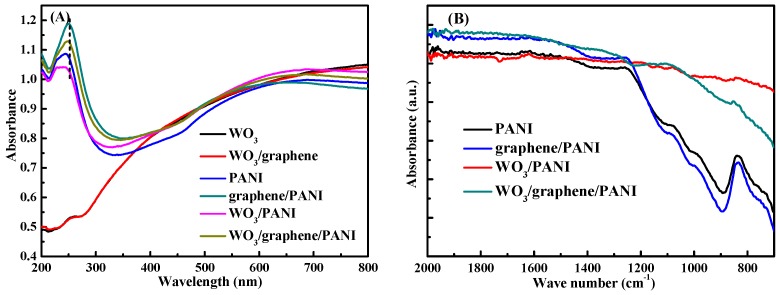
(**A**) UV-vis and (**B**) FT-IR spectra of bare WO_3_ film, double layer WO_3_/graphene film, pristine PANI film, double layer graphene/PANI film, double layer WO_3_/PANI film, and triple layer WO_3_/graphene/PANI film, respectively.

In order to characterize the relationships between nanofillers and PANI matrix as well as the inherent composition of the nanocomposites, UV-Vis and FT-IR are conducted and shown in Figure 2 as bare WO_3_, double layer WO_3_/PANI, pristine PANI, double layer graphene/PANI, double layer WO_3_/PANI, and triple layer WO_3_/graphene/PANI. Figure 2A shows the UV-vis conducted in the wavelength range of 200–800 nm. The characteristic peak of PANI centering around 240 nm is missing for bare WO_3_ and double layer WO_3_/graphene films compared with that of the PANI-based ones, indicating the successful deposition of PANI in the other four films. Furthermore, a clear shift of the characteristic peak is observed for all the PANI-based nanocomposites, indicating the existence of chemical bonds between the PANI matrix and WO_3_ particles. The nitrogen atoms in PANI are inferred to possibly form coordinated compounds with the exposed tungsten atoms on the surface of WO_3_. The strong coordination bonds between the nitrogen atom and the tungsten atom might cause the observed peak shifts. FT-IR as a strong technique probing the inherent composition and inner relationships of the PANI-based nanocomposites is carried out and shown in Figure 2B. Even though the amounts of PANI on the tested area for each film are relatively too low to show strong enough signals, all the FT-IR spectra display similar peaks around 900, 1000, and 1100 cm^−1^, corresponding to the characteristic peaks of PANI [21,22]. Moreover, the strength of FT-IR peaks in Figure 2B shows an obvious trend of graphene/PANI > PANI > WO_3_/graphene/PANI > WO_3_/PANI, which is consistent with the PANI amounts calculated above.

**Figure 3:**
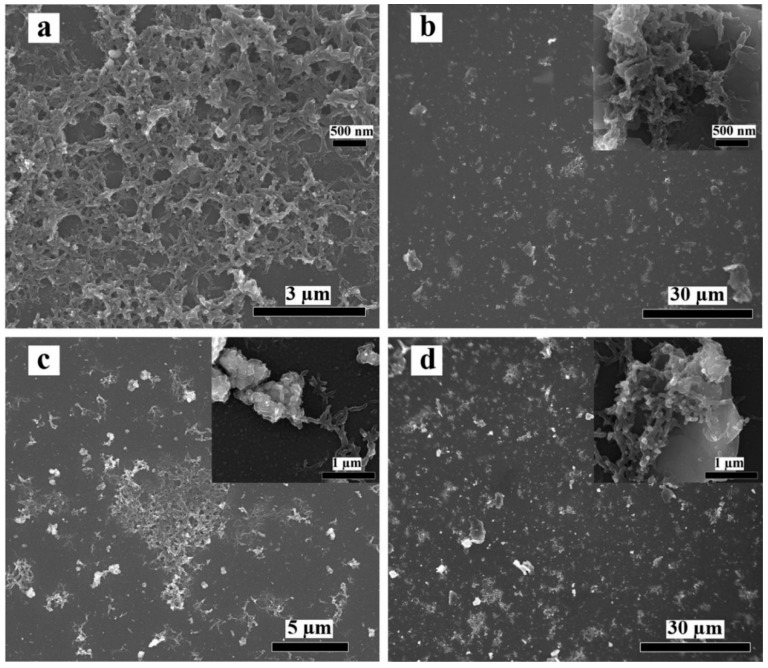
SEM images of (**a**) pristine PANI film, (**b**) graphene/PANI film, (**c**) WO_3_/PANI film, and (**d**) WO_3_/graphene/PANI film. Inset is the magnification of corresponding SEM image.

The morphologies of the synthesized hybrid films have been characterized by SEM as shown in Figure 3, which represents the SEM images of (a) pristine PANI, (b) graphene/PANI, (c) WO_3_/PANI, and (d) WO_3_/graphene/PANI films, respectively. The high magnifications of the corresponding SEM images are shown as the insets. For pristine PANI, Figure 3a, the short fiber-liked PANI is successfully deposited on the ITO glass slide through this electro-polymerization method and form a porous PANI matrix. The PANI matrix is also clearly confirmed by the magnification of this SEM image shown as inset in Figure 3a. Various morphologies of PANI can be obtained by adjusting the conditions of the electrochemical polymerization process, which will further affect the electrochemical performance of the PANI-based films. Nevertheless, the porous PANI network made by nanofibrous PANI is capable to provide not only high surface area but also transmission channels for electrons, which is supposed to be beneficial for the electrochemical performance of the PANI-based films. For the graphene/PANI film, Figure 3b, graphene sheets and PANI matrix are both seen and uniformly distributed on the ITO glass. Furthermore, a closer insight shows clear interactions between the PANI fibers and graphene sheets shown as inset in Figure 3b. Similarly, for WO_3_/PANI, Figure 3c, WO_3_ nanoparticles and PANI matrix are also uniformly distributed on the ITO glass with clear interaction between WO_3_ particles and PANI fibers, which is also confirmed from the magnification of Figure 3c. Finally, for the triple layer WO_3_/graphene/PANI, Figure 3d, both graphene sheets and WO_3_ particles are clearly seen to distribute in the PANI matrix, the interactions are also confirmed by the close wrapping of PANI fibers on graphene sheets and WO_3_ particles shown as inset in Figure 3d. The interactions between the PANI matrix and nanofillers are consistent with the FT-IR and UV-vis analysis. In addition, the morphologies and the layer thickness are also investigated by AFM as shown in Figure S1; below of each is the height profile of the corresponding film. For bare WO_3_ film, Figure S1a, the thickness is not a constant value because of the random distribution of WO_3_ nanoparticles. In contrast, other films have constant thickness values based on the corresponding profiles. The thickness of the synthesized PANI-based films follows an order as WO_3_/graphene/PANI (~33 nm) > WO_3_/PANI (~30 nm) > graphene/PANI (~22 nm) > pristine PANI (~20 nm), which is consistent with the composition of the synthesized films.

### 3.3. Electrochromic Behaviors

**Figure 4:**
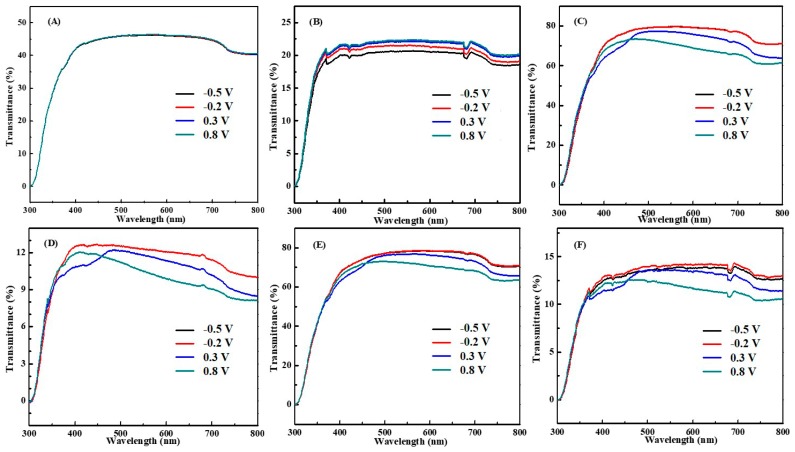
UV-vis spectra of (**A**) pure WO_3_ film, (**B**) double layer WO_3_/graphene film, (**C**) pristine PANI film, (**D**) double layer graphene/PANI film, (**E**) double layer WO_3_/PANI film, and (**F**) triple layer WO_3_/graphene/PANI film in 0.5 M H_2_SO_4_ aqueous solution at different potentials as −0.5, −0.2, 0.3, and 0.8 V.

Figure 4 shows the UV-vis transmission spectra of (A) pure WO_3_ film, (B) double layer WO_3_/graphene film, (C) pristine PANI film, (D) double layer graphene/PANI film, (E) double layer WO_3_/PANI film, and (F) triple layer WO_3_/graphene/PANI film at different potentials (−0.5, −0.2, 0.3 and 0.8 V) in 0.5 M H_2_SO_4_, respectively. For all the PANI-based nanocomposites with applied potential from −0.5 to 0.8 V (Figure 4C–F) similar UV-vis spectra can be clearly observed as the transmittance decreases continuously with the increasing potential, indicating the promoted oxidation of PANI. In addition, the characteristic absorbance band from 500 to 750 nm belongs to the emeraldine salt form in PANI resulting from the π–π* transition of the quinoid ring, especially for the highly oxidized state at 0.8 V, implying the dominant role of PANI in the hybrid PANI-based films [44]. For bare WO_3_ film, Figure 4A shows that the transmittance keeps constant with varying the potential because of the small amount of WO_3_ and the narrow potential range of WO_3_. The transmittance of double layer WO_3_/graphene film exhibits small increased transmittance values with increasing potential from −0.2–0.8 V, which can be attributed to the altered electronic structure of the introduced graphene. However, it is noticeable that the transmittance values decrease largely after the introduction of graphene into WO_3_ film compared with that of bare WO_3_ film, which can be ascribed to the block role of graphene. Similar phenomenon is also observed in Figure 4D after introducing graphene into the pristine PANI film, the transmittance values at different potentials remain almost the same with slightly decreasing trend because of the light block effect of graphene. However, no obvious difference was observed in Figure 4E for WO_3_/PANI film compared with that of pristine PANI film. Finally, it is clearly seen from Figure 4F that all the transmittance values of the triple layer WO_3_/graphene/PANI film are relatively low (~15%) because of the introduced graphene. However, the film also exhibits obvious decreased transmittance values and typical PANI peaks with the increasing potentials. 

**Figure 5:**
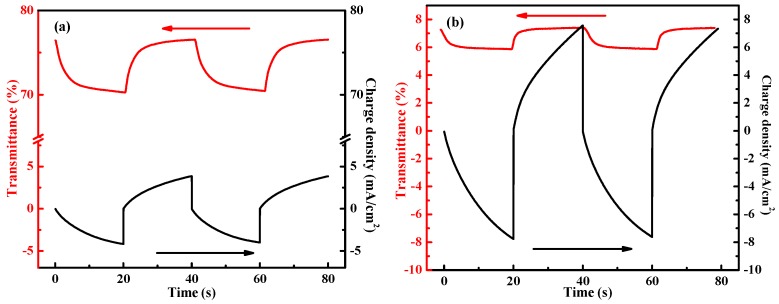

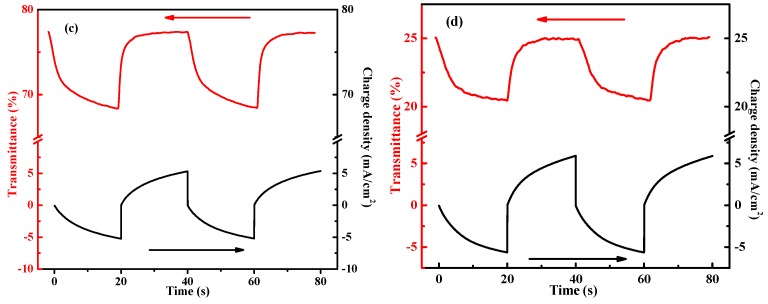
In situ transmittance (red) and corresponding chronocoulometry (black) of (**a**) pristine PANI film, (**b**) graphene/PANI film, (**c**) WO_3_/PANI film, and (**d**) WO_3_/graphene/PANI film at 633 nm in 0.5 M H_2_SO_4_ aqueous solution. The tests were conducted under a square-wave voltage of 0.8 and −0.2 V with a pulse width of 20 s.

The coloration switching responses as an important parameter is vital to evaluate the electrochromic performance in terms of energy consumption and coloration switching. The coloration properties of all the PANI-based nanocomposites films were studied by applying potential steps of 0.8 and −0.2 V with a pulse width of 20 s. Figure 5a–d shows the transmittance-time (red) and the corresponding charge density-time curves (black) at 633 nm under an alternative square-wave voltage of 0.8 and −0.2 V with 20 s interval. The transmittance modulations, transmittance difference between the bleached and colored states in the electrochromic materials, as 6.12, 1.71, 9.04, and 4.52% are calculated for the pristine PANI, double layer graphene/PANI, double layer WO_3_/PANI, and triple layer WO_3_/graphene/PANI nanocomposites films, respectively. Decreased transmittance modulations of graphene contained films confirm further the block effect of the introduced graphene.

Coloration efficiency (CE or *η*) is a critical parameter of electrochromic materials for practical applications. It is defined as the change in the optical density (OD) per unit charge (Q) inserted into (or extracted from) the electrochromic films, that is, the amount of energy to achieve color change. The CE is calculated from Equations (2) and (3):

*η* = Δ*OD*(*λ*)/*Q_d_* (2)

**Figure 6:**
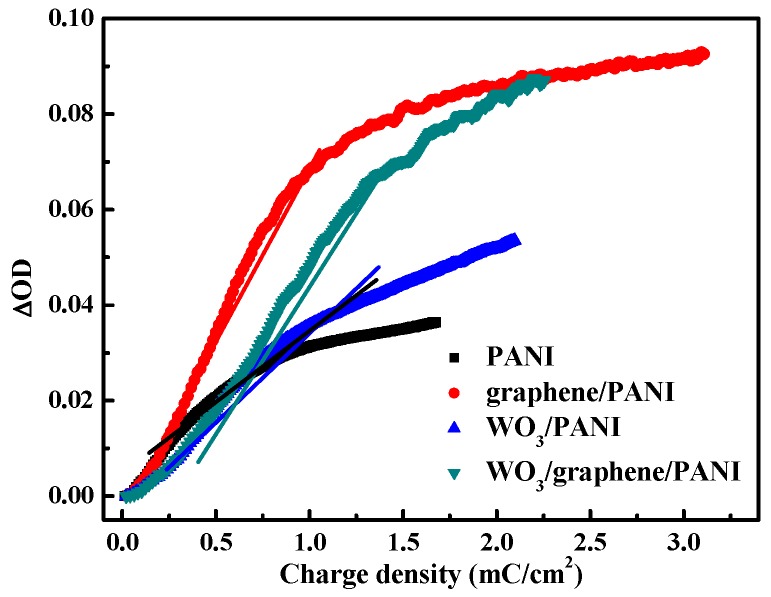
The plots of in situ optical density (ΔOD) versus charge density of pristine PANI film, graphene/PANI film, WO_3_/PANI film, and WO_3_/graphene/PANI films. The optical density was measured at 633 nm at 0.8 V in 0.5 M H_2_SO_4_ aqueous solution.

Δ*OD* = log[*T_colored_*/*T_bleached_*] (3)

where Δ*OD* is the change in the optical density, *λ* is the dominant wavelength for the material, *Q_d_* is the charge density (injected/ejected charges per unit electrode area), *T_beleached_* refers to the transmittance of the film in the bleached state, and *T_colored_* refers to the varying transmittance of the film during the coloring process. Figure 6 depicts the plots of the calculated Δ*OD* from the second cycle in the transmittance-time curve at 633 nm versus the corresponding inserted charge density obtained from charge density-time curve (Figure 5). The *η* is extracted as the slope of the straight line fitting to the linear region of the curve. The *η* values of composite films are all found to be larger than that of pristine PANI and increasing follow an order as graphene/PANI > WO_3_/graphene/PANI > WO_3_/PANI > pristine PANI. The improved CE for the PANI-based nanocomposites films indicate an enhanced color changing response ability as fewer injected charges result in larger optical density change than that of the pristine PANI film. The films with graphene exhibit a greatly increased *η* due to the largely decreased resistance and properly formed matrix structure. Furthermore, the enhanced *η* value of WO_3_/graphene can be attributed to the resulted porous nanostructures from the WO_3_ embedment. 

### 3.4. Capacitive Energy Storage Performances

**Figure 7:**
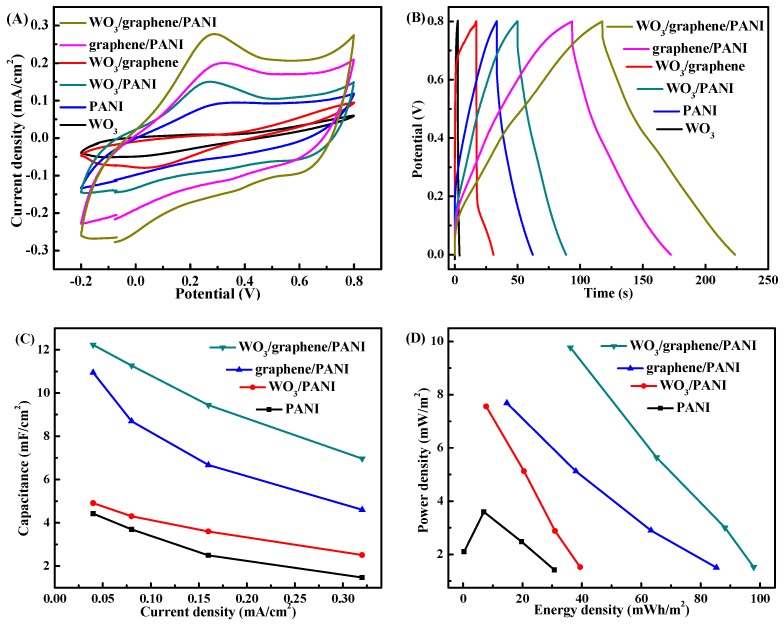
(**A**) CVs conducted at a scan rate of 50 mV/s, (**B**) charge-discharge curves with a current density of 0.08 mA/cm^2^, (**C**) specific capacitances depended on current density, and (**D**) Ragone plots of bare WO_3_ film, double layer WO_3_/graphene film, pristine PANI film, double layer WO_3_/PANI film, double layer graphene/PANI film, and triple layer WO_3_/graphene/PANI film measured in 0.5 M H_2_SO_4_ aqueous solution.

The capacitive performances of the different films were studied by cyclic voltammogram (CV) at a high scan rate of 50 mV/s within a potential range of −0.2 to 0.8 V in 0.5 M H_2_SO_4_ as shown in Figure 7A. The CV curves of the PANI-based nanocomposites films at other scan rates (100, 20, and 5 mV/s) are also provided in Figure S2. Higher current densities with larger enclosed CV areas are clearly seen in WO_3_/graphene/PANI, followed by graphene/PANI, WO_3_/PANI, pristine PANI, WO_3_/graphene, and bare WO_3_, indicating more energy stored in the PANI-based films after introducing the corresponding nanofillers because of the increased PANI amount and the capacitance contributions of the nanofillers. Furthermore, Figure 7A shows that all the PANI-based nanocomposites exhibit nonrectangular CV curves with obvious one pair of redox peaks around 0.4/0.3 V, which are characteristic peaks of PANI oxidation and reduction. However, it is also noted that the oxidation peaks have slight shift because of the embedment of the nanofillers, which confirms the proper interactions between PANI matrix and nanofillers. Obviously, the WO_3_/graphene/PANI exhibits a much higher current density than other film counterparts, suggesting that WO_3_/graphene/PANI film is the most promising one for supercapacitor application among all the samples. 

Galvanostatic charge-discharge (GCD) as a reliable approach to measure the specific capacitance of supercapacitor is also carried out in the same H_2_SO_4_ solution from 0 to 0.8 V with a current density of 0.08 mA/cm^2^, as shown in Figure 7B. All the GCD curves of PANI-based nanocomposites exhibit non-straight lines because of the dominant pseudo-capacitor role of PANI. The increasing discharging time always represents a higher capacitance. It is observed that the capacitance of the pristine PANI film can be greatly enhanced through introducing WO_3_ and graphene nanofillers, confirmed by the discharging time order as pristine PANI < WO_3_/PANI < graphene/PANI < WO_3_/graphene/PANI, which is also in good accordance with the area of CV results. In addition, the voltage drop at the initiation of the discharge curve is extremely small for WO_3_/graphene/PANI among all the films, indicating a very low equivalent series resistance (ESR) in the supercapacitor and also enables high-power operations. The specific capacitances of different films calculated from the CV at 50 mV/s and the GCD at 0.08 mA/cm^2^ are summarized in Table 1, which follow the same order as WO_3_/graphene/PANI > graphene/PANI > WO_3_/PANI > pristine PANI > WO_3_/graphene > bare WO_3_, confirming the positive effects of graphene and WO_3_. Furthermore, the correlation between the specific capacitance and the current density for these four PANI-based films is also presented in Figure 7C. The WO_3_/graphene/PANI has the highest capacity among all samples at all the same scan rates. For instance, the WO_3_/graphene/PANI achieves a specific capacitance of 11.267 mF/cm^2^ with 0.08 mA/cm^2^ current density, which is 1.3 and 2.6 times higher than that of graphene/PANI (8.709 mF/cm^2^) and WO_3_/PANI (4.312 mF/cm^2^), respectively. Notably, WO_3_/graphene/PANI shows capacitance three times larger than that of pristine PANI (3.693 mF/cm^2^), indicating a dramatic capacitance increasement when employing graphene and WO_3_ nanofillers. Furthermore, the triple layer WO_3_/graphene/PANI film shows a capacity of 12.27 and 7.017 F/g with 0.04 and 0.32 mA/cm^2^ current density, respectively, suggesting a considerable rate capability of WO_3_/graphene/PANI. The improvement in power density of WO_3_/graphene/PANI is further confirmed by the Ragone plots of these four PANI-based films as shown in Figure 7D. Similar power density trend was clearly observed as WO_3_/graphene/PANI > graphene/PANI > WO_3_/PANI > pristine PANI as a function of energy density. Furthermore, the as-developed WO_3_/graphene/PANI can outperform a lot of reported mass-based values in terms of capacitance and energy density [23,31,32]. It is noted that, besides the appealing electrochemical properties as supercapacitor, this facile prepared WO_3_/graphene/PANI film may also show considerable performances regarding PANI related practical applications like chemical vapor sensors, transparent conductors, and actuators.

**Figure 8:**
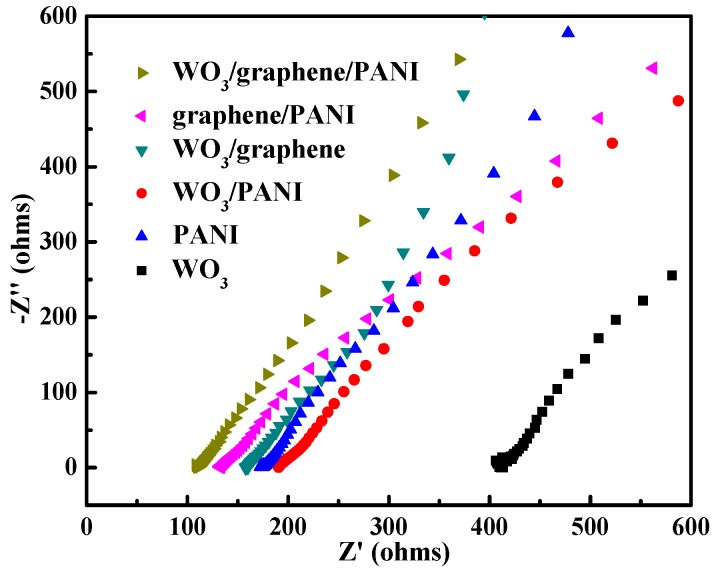
Nyquist plots of bare WO_3_, WO_3_/graphene, pristine PANI, WO_3_/PANI, graphene/PANI, and WO_3_/graphene/PANI films in 0.5 M H_2_SO_4_ aqueous solution, respectively.

Electrochemical impedance spectroscopy (EIS) as a power technique probing the inherent reaction kinetics of the electrode was also employed as shown in Figure 8. The semicircular Nyquist plots of imaginary (Z″, Ω) versus real (Z′, Ω) components of impedance were conducted in the frequency range of 100,000 to 0.01 Hz with a 5 mV amplitude referring to open potential in 0.5 M H_2_SO_4_. It is clearly seen that the resistance of bare WO_3_ film is larger than the others throughout the mostly frequency region due to the low conductivity of WO_3_. Meanwhile, the decreased resistance of WO_3_/PANI and WO_3_/graphene results from the introduced highly conductive PANI and graphene, respectively. However, no resistor-capacitor (RC) loops or semicircles appear in the high frequency region for PANI-based films, indicating negligible charge resistances and the better energy storage properties of the PANI-based films. The excellent energy storage performance of WO_3_/graphene/PANI can be clearly demonstrated as the tail approaching an almost vertical line compared with others. In addition, the equivalent series resistance (ESR) of each sample can be calculated from the Z′ values at Z″ = 0, which has been shown in Table S1. The resistivity of the films follows the order as WO_3_/graphene/PANI < graphene/PANI < WO_3_/graphene < pristine PANI < WO_3_/PANI < WO_3_, demonstrating enhanced reaction kinetics because of the positive effects of graphene and WO_3_ nanofillers, which is also consist with the CV and GCD analysis. 

### 3.5. Cycling Stability

**Figure 9:**
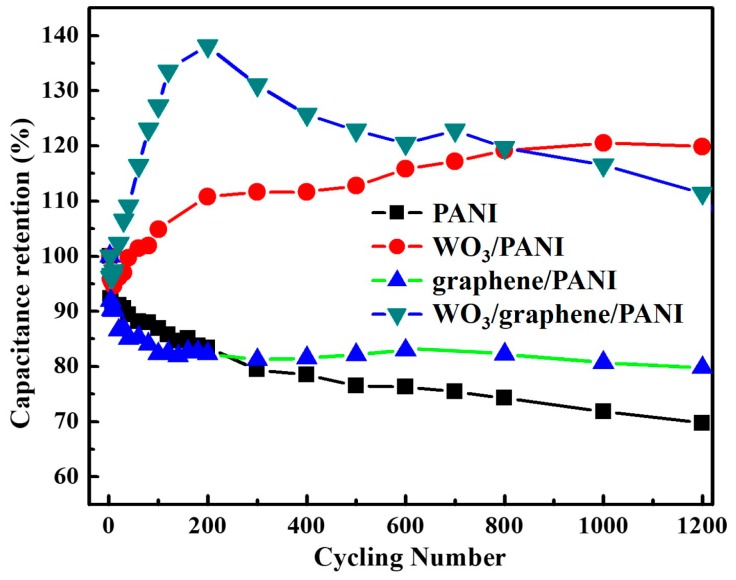
Cycling stability of pristine PANI, WO_3_/PANI, graphene/PANI, and WO_3_/graphene/PANI film electrodes at 0.16 mA/cm^2^ for 1200 cycles in 0.5 M H_2_SO_4_ aqueous solution.

Since a long cycling performance is of the most important criteria for supercapacitors, the cycling performances of these four PANI-based films were conducted by GCD for 1200 cycles with a current density of 0.16 mA/cm^2^. The cycling performances with specific capacitance retention are shown in Figure 9. It is clearly seen that all the PANI-based nanocomposites films exhibit much better cycling performance than that of pristine PANI. For example, the graphene/PANI has an 80% retention, higher than that of pristine PANI (70%), indicating the positive role of graphene which is also consistent with reported works [45,46]. However, both the WO_3_/graphene and WO_3_/graphene/PANI electrodes have slight capacitance increases during the cycling process. The capacitance increases can be attributed to the introduced WO_3_ nanoparticles, which were increasingly accessed along with cycling. The WO_3_ particles used in this work have a nanoscale size around 40–50 nm, indicating a high specific surface area of the nanoparticles. Therefore, a combined electric double-layer capacitance from high surface area and pseudo-capacitor capacitance from redox reactions can be delivered by WO_3_ nanoparticles. Accompanied by the charge-discharge cycles, the activation and volume change of WO_3_ nanoparticles lead to higher pseudo-capacitor capacitance from more redox active sites, that is, enhanced capacitance during cycling. The cycling performance of the as-prepared PANI films follows the order as WO_3_/PANI > WO_3_/graphene/PANI > graphene/PANI > pristine PANI, which implies the WO_3_/graphene/PANI film is capable to serve as a promising supercapacitor material because of its much-enhanced capacitance and cycling performance. The above results clearly reveal the potential applications of this triple layer WO_3_/graphene/PANI for supercapacitors, while also highlighting again the positive effects of WO_3_ and graphene nanofillers on the PANI matrix. 

## 4. Conclusions

Different hybrid WO_3_, graphene, and PANI films have been successfully prepared by electrodepositing PANI monomers onto pre-spincoated WO_3_, graphene, and WO_3_/graphene ITO glasses. Multi-color electrochromic phenomenon and supercapacitive performance of the different PANI films have been observed in this nanocomposite film owing to the dominant PANI component in the composites. Higher coloration efficiency and faster switching responses of the PANI-based nanocomposites films than those of the pristine PANI film are obtained from the inner interactions between the PANI matrix and the nanofillers, as well as the resulted rougher morphology. However, the introduced graphene can decease the light transmittance modulation of the PANI-based films because of its light block effect. The PANI-based nanocomposites films also exhibit enhanced areal capacitances compared to that of the pristine PANI film because of the capacitive role of nanofillers and the increased deposition amount of PANI materials. Largely enhanced cycling stabilities were also obtained because of the positive effects of graphene and WO_3_. In summary, this novel triple layer WO_3_/graphene/PANI nanocomposites film, incorporating the advantages of both graphene and WO_3_ in PANI matrix, has exhibited promising application potential in devices integrated the functions of both electrochromic and energy storage because of its considerable electrochromic and capacitive behaviors.

## Figures and Tables

**Table 1:** Summary of capacitance, energy density, and power density values of the different films calculated for cyclic voltammogram (CV) and galvanostatic charge-discharge (GCD) results (Calculation methods shown in supporting materials).

Film	CV C_S_. 50 mV/s (mF cm^−2^)	GCD Cs. 0.08 mA/cm^2^ (mF cm^−2^)	Energy Density (mWh/m^2^)	Power Density (mW/m^2^)
WO_3_	0.489	0.253	0.688	1.770
WO_3_/graphene	0.764	2.586	6.360	1.683
PANI	1.371	3.693	19.69	2.478
WO_3_/PANI	1.856	4.312	30.95	2.879
graphene/PANI	2.629	8.709	63.29	2.895
WO_3_/graphene/PANI	3.414	11.267	88.17	3.003

## Supplementary Materials

The following are available online at https://www.mdpi.com/2073-4360/12/1/49/s1. Figure S1: AFM images of (**a**) bare WO_3_ film, (**b**) double layer WO_3_/graphene film, (**c**) pristine PANI film, (**d**) double layer graphene/PANI film, (**e**) double layer WO_3_/PANI film, and (**f**) triple layer WO_3_/graphene/PANI film. Below of each is the height profile of the corresponding film. Figure S2: CV (**A**, **C**, **E**, and **G**) and GCD (**B**, **D**, **F**, and **H**) curves of (A and B) pristine PANI, (C and D) WO_3_/PANI, (E and F) graphene/PANI, and (G and H) WO_3_/graphene/PANI in 0.5 M H_2_SO_4_ with different CV scan rates as (a) 100, (b) 50, (c) 20, and (d) 5 mV/s and different GCD current densities as (a) 0.32, (b) 0.16, (c) 0.08, and (d) 0.04 mA/cm^2^. Table S1: The equivalent series resistance (ESR) values of the different films calculated from the Nyquist plots.
