# Supplementary material for: Triple Layer Tungsten Trioxide, Graphene, and Polyaniline Composite Films for Combined Energy Storage and Electrochromic Applications
Source: Polymers (Basel). 2019 Dec 30;12(1):49. doi: 10.3390/polym12010049 (PMC7023535; doi:10.3390/polym12010049)
Supplement: Supplementary file 1 [file polymers-12-00049-s001.pdf]

Triple Layer Tungsten Trioxide, Graphene, and Polyaniline Composite Films for Combined Energy Storage and Electrochromic Applications

Hailong Lyu[ab*]

[a] Chemical Sciences Division, Oak Ridge National Laboratory, Oak Ridge, TN 37831, USA.

[b] Department of Chemistry, University of Tennessee, Knoxville, TN 37996, USA

*Author to whom correspondence should be addressed.

E-mail: hlyu@utk.edu (H. L.)

Calculations of Capacitance, Energy Density and Power Density

The capacitance of the electrodes was calculated from the corresponding CV curves at different scan rates from the corresponding CV curves at different scan rates from 0 to 1 V in 0.5 M H_2SO_4 aqueous solution using Equation (1):

$$C_s = (\int idV) \Big/ (2m \times \Delta V \times \nu) \qquad (1)$$

Where C_s is the specific capacitance in F/g, $\int idV$ is the integrated area of the CV curve, *m* is the mass of the active materials in the single electrode in *g*, ΔV is the scanned potential window in V, and ν is the scan rate in V/s.

The capacitance from the GCD curves was calculated using Equation (2):

$$C_s = (i \times t) / (m \times \Delta V) \qquad (2)$$

Where C_s is the specific gravimetric capacitance in F/g, *i* is the discharge current in A, *t* is the discharge time in *s*, *m* is the mass of the active materials on the working electrode in *g*, and ΔV is the scanned potential window in V (excluding the IR drop at the beginning of the discharge process).

The energy density, *E*, and power density, *P*, of the electrode materials were calculated from Equations (3) and (4), respectively,

$$E = \frac{C_s \Delta V^2}{7.2} \qquad (3)$$

$$P = \frac{3600E}{t} \qquad (4)$$

where *E* is the specific energy density in Wh/kg, *P* is the specific power density in W/kg, C_s is the

specific capacitance in F/g, ΔV is the scanned potential window (excluding IR drop at the beginning of the discharge process) in V, and t is the discharge time in s.

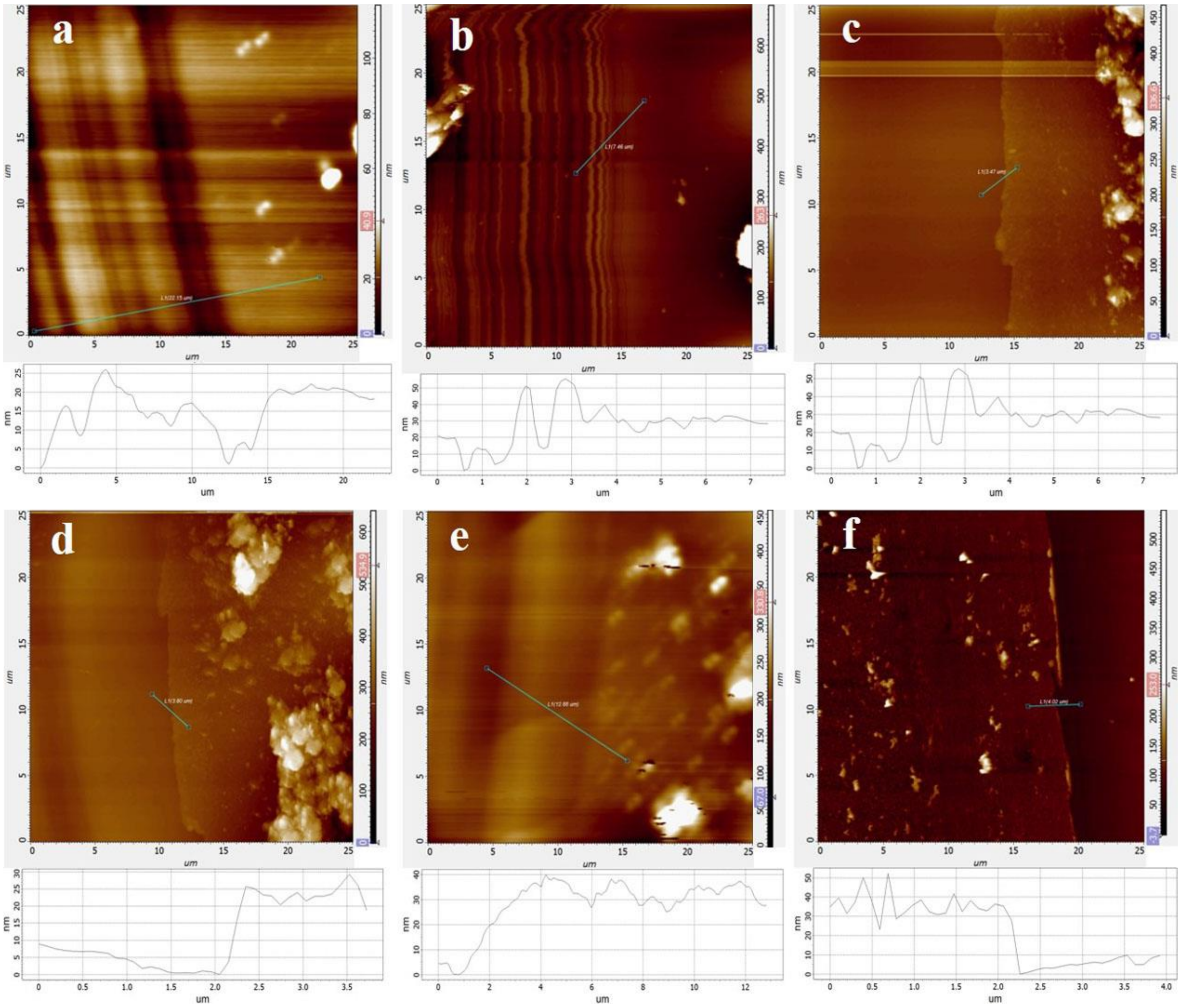

Figure S1. AFM images of (a) bare WO_3 film, (b) double layer WO_3/graphene film, (c) pristine PANI film, (d) double layer graphene/PANI film, (e) double layer WO_3/PANI film, and (f) triple layer WO_3/graphene/PANI film. Below of each is the height profile of the corresponding film.

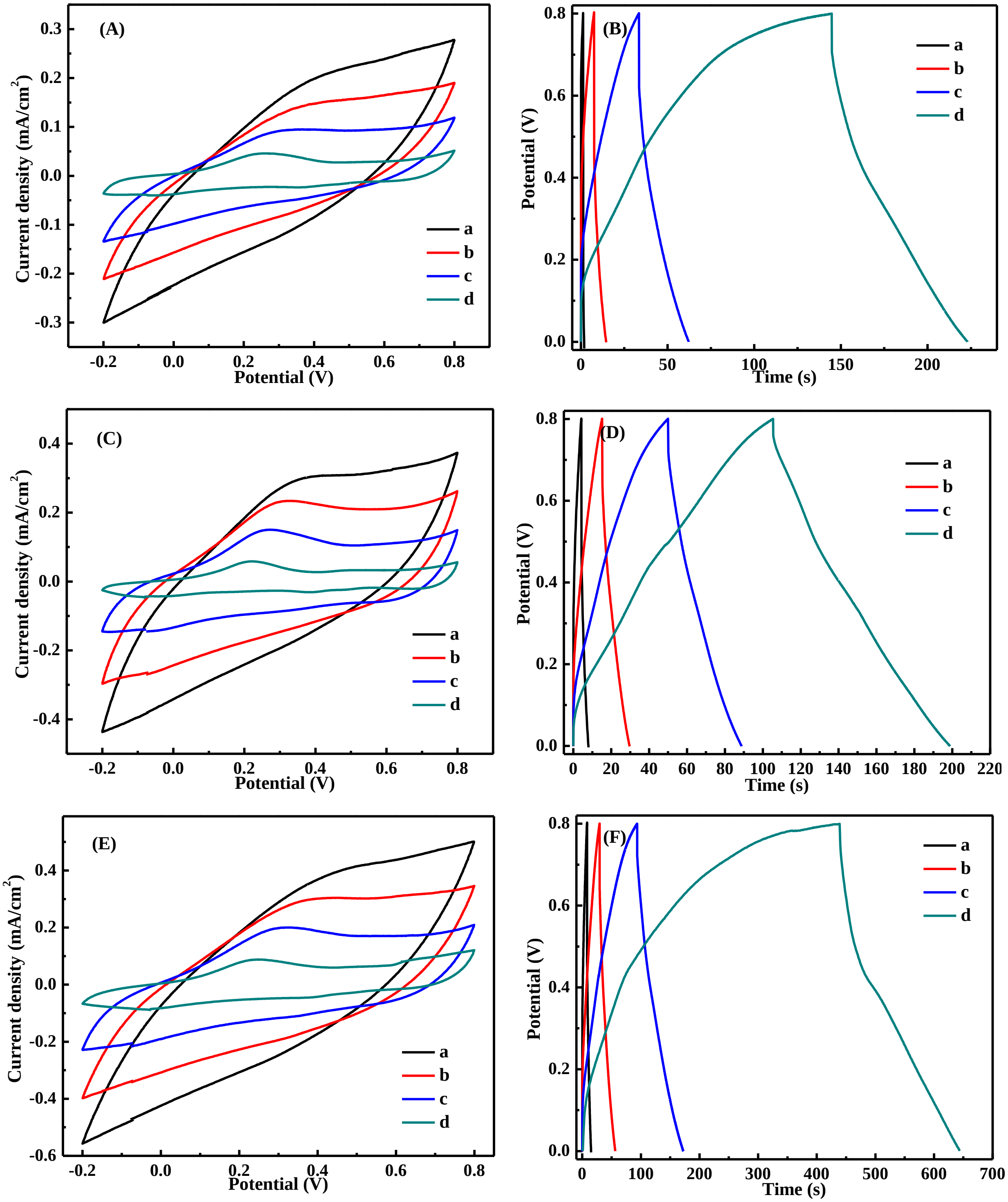
(A)
Current density (mA/cm^2)
Potential (V)
a
b
c
d
(B)
Potential (V)
Time (s)
a
b
c
d
(C)
Current density (mA/cm^2)
Potential (V)
a
b
c
d
(D)
Potential (V)
Time (s)
a
b
c
d
(E)
Current density (mA/cm^2)
Potential (V)
a
b
c
d
(F)
Potential (V)
Time (s)
a
b
c
d

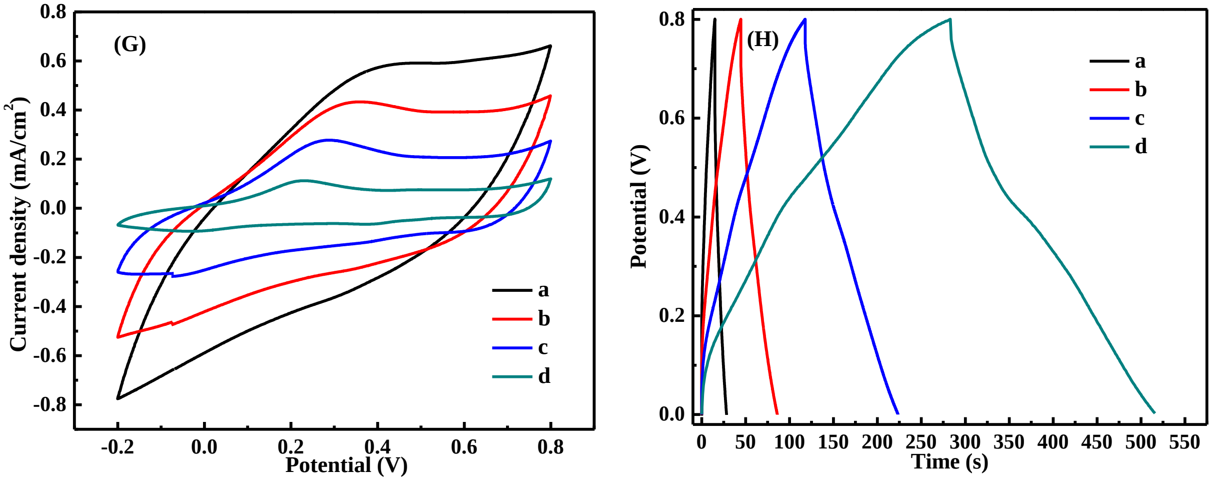

Figure S2. CV (A, C, E and G) and GCD (B, D, F and H) curves of (A&B) pristine PANI, (C&D) WO3/PANI, (E&F) graphene/PANI, and (G&H) WO3/graphene/PANI in 0.5 M H_2SO_4 with different CV scan rates as (a) 100, (b) 50, (c) 20, and (d) 5 mV/s and different GCD current densities as (a) 0.32, (b) 0.16, (c) 0.08, and (d) 0.04 mA/cm^2

Table S1. The equivalent series resistance (ESR) values of the different films calculated from the Nyquist plots.

Film	Resistivity (Ω)
WO_3	406.2
PANI	172.4
WO_3/PANI	189.8
WO_3/graphene	157.6
graphene/PANI	131.8
WO_3/graphene/PANI	108.4
